# Effect of Sericin Content on the Structural Characteristics and Properties of New Silk Nonwoven Fabrics

**DOI:** 10.3390/biom13081186

**Published:** 2023-07-29

**Authors:** Ye Eun Kim, Yu Jeong Bae, Mi Jin Jang, In Chul Um

**Affiliations:** 1 Department of Biofibers and Biomaterials Science, Kyungpook National University, Daegu 41566, Republic of Koreaqodbwjd01@naver.com (Y.J.B.); 2 Preclinical Research Center, Daegu-Gyeongbuk Medical Innovation Foundation, Daegu 41061, Republic of Korea

**Keywords:** sericin content, silk nonwoven fabric, carding, structural characteristics, mechanical properties, cell viability

## Abstract

Recently, natural silk nonwoven fabrics have attracted attention in biomedical and cosmetic applications because of their excellent biocompatibility, mechanical properties, and easy preparation. Herein, silk nonwoven fabrics were prepared by carding silk filaments to improve their productivity, and the effect of sericin content on the structure and properties of silk nonwoven fabrics was investigated. Owing to the binding effect of sericin in silk, a natural silk nonwoven fabric was successfully prepared through carding, wetting, and hot press treatments. Sericin content affected the structural characteristics and properties of the silk nonwoven fabrics. As the sericin content increased, the silk nonwoven fabrics became more compact with reduced porosity and thickness. Further, with increasing sericin content, the crystallinity and elongation of the silk nonwoven fabrics decreased while the moisture regain and the maximum stress increased. The thermal stability of most silk nonwoven fabrics was not affected by the sericin content. However, silk nonwoven fabrics without sericin had a lower thermal decomposition temperature than other nonwoven fabrics. Regardless of the sericin content, all silk nonwoven fabrics exhibited optimal cell viability and are promising candidates for cosmetic and biomedical applications.

## 1. Introduction

Silk is a naturally occurring biomaterial composed of fibroin and sericin. It is biodegradable [1,2] and has excellent biocompatibility [3,4,5], excellent cell adhesion and proliferation [6,7,8], high water retention capacity [9], and wound healing effect [10,11]. Due to these advantageous properties, silk has attracted attention in biomedical and cosmetic applications [12,13].

For these applications, silk must be prepared into various forms, including sponge [14,15], film [16,17], gel [18,19,20], fiber [21,22,23], web [24,25], nonwoven fabric [26,27,28], and bead/particle [29,30]. Among these, the porous silk forms (e.g., sponge and web) hold fluids and provide spaces where cells can proliferate [31,32,33]. Consequently, porous silk forms have been studied for membrane applications for guided bone regeneration [34,35,36], nerve conduit [37], bone substitute [38], wound dressing [10,11,39], drug delivery [40], and mask pack [41,42].

In fabricating porous silk materials, the electrospinning technique has been extensively employed. However, it requires several preparation steps (e.g., degumming, drying, dissolution, dialysis, and electrospinning) and is time-consuming [24,25,43]. Furthermore, the high crystallinity and molecular weight of silk are affected during the regeneration process (i.e., degumming and dissolution), resulting in poor mechanical properties of silk [44,45,46].

Recently, natural silk nonwoven fabrics were prepared by utilizing the binding character of sericin [27,28,47,48,49] to eliminate the drawbacks of electrospun regenerated silk webs. In this method, silk fibers were reeled from silkworm cocoons to form a silk web. Although natural silk nonwoven fabrics exhibit excellent mechanical properties [27,28,49], if silk fibers are broken during reeling, the reeling (winding) process becomes economically inefficient. In addition, a reeling bath at elevated temperatures (>50 °C) is necessary to reel the silk fibers [50].

Meanwhile, in the conventional mass production of nonwoven fabrics, dry fibers are arranged to prepare a web following the carding process. In this method, the webs can be mass-produced with no breakage of fibers and no requirement of additional equipment (heating bath), making the process economically efficient. However, although the preparation of new natural silk nonwoven fabrics using the reeling process has been studied, the new silk nonwoven fabrics using the binding character of sericin have not been prepared by arranging silk fibers with a carder.

Herein, raw silk filaments and silk filaments degummed with different methods (i.e., with varying sericin content) were prepared. Subsequently, they were carded, wet-treated, and hot-pressed to prepare natural silk nonwoven fabrics. In addition, the effects of sericin content in silk filaments on the structural characteristics and properties of the fabrics were investigated.

## 2. Materials and Methods

### 2.1. Materials

*Bombyx mori* Baekokjam silkworm cocoons were provided by the National Institute of Agricultural Science (Wanju, Republic of Korea). The silkworm cocoons were dried for 4 h at a high temperature (90 °C) to kill the pupa.

### 2.2. Preparation of Silk Fibers with Varying Sericin Contents

Initially, silkworm cocoons were immersed in a 70% (*v*/*v*) ethanol aqueous solution at 50 °C for 2 d to remove nonprotein components (e.g., wax, carbohydrates, organic matter, and pigment). The ratio of the cocoon and ethanol aqueous solution was 1:30 (*w*/*v*). Then, the silkworm cocoons were washed with purified water and then dried at room temperature. Consequently, 2.0% (±0.2%) of nonprotein components for the whole weight of the cocoon were removed from the cocoons, which are determined by the weight change of the cocoon between before and after the 70% ethanol solution treatment.

Silk fibers with varying sericin contents (0–24%) were obtained by controlling the degumming conditions (i.e., degumming method and time), as shown in Table 1. *Bombyx mori* silkworm cocoons were degummed by the high-temperature high-pressure (HTHP) method or the soap/soda method. The degumming ratio was calculated using Equation (1) [51,52]:

(1) $$Degumming\;ratio\;(\%)=\left(1-\frac{W_{1}}{W_{2}}\right)\times 100$$

where $W_{1}$ denotes the dry mass of the degummed cocoons and $W_{2}$ denotes the dry mass of the cocoons.

Since the sericin content of the silkworm cocoon used herein was 24% [53], the content was calculated using Equation (2):

(2) Sericin content (%)=[1−{(1−0.24)/(1−degumming ratio)}]×100

The HTHP method was employed to prepare silk fibers with sericin contents of 3.2%–21.5%. That is, the silk was degummed using purified water at 120 °C for 1, 3, 15, 22, and 30 min using an autoclave (JSAC-60, JSR, Gongju, Republic of Korea). The cocoon-to-water ratio was 1:50 (*w*/*v*) [54]. Meanwhile, the soap/soda method was employed to remove sericin completely from silk to prepare silk fibers with 0% sericin (i.e., fibroin). The silkworm cocoons were degummed in a boiling aqueous solution containing 0.3% (*w*/*v*) sodium oleate and 0.2% (*w*/*v*) sodium carbonate at 100 °C for 1 h. The cocoon-to-solution ratio was 1:25 (*w*/*v*). The degummed silk was washed with purified water and dried at 105 °C for 24 h. Purified water was obtained using a water purification system (RO50, Hana Science, Hanam, Republic of Korea) with a reverse osmosis membrane. The preparation conditions of the silk fibers with varying sericin contents are summarized in Table 1.

### 2.3. Preparation of Silk Nonwoven Fabrics

**Figure 1:**
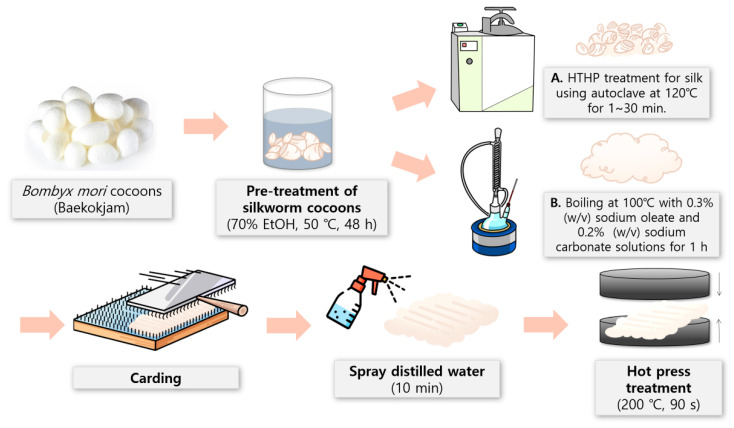
Schematic of the preparation process of silk nonwoven fabrics with varying sericin contents.

The preparation process of silk nonwoven fabric is depicted in Figure 1. First, the silk fibers with varying sericin contents (0–24%) were carded (arranged) using a lab-scale hand carder and a blending board (Standard Hand Cards, Brother Drum Carder, Silverton, CO, USA). One gram of silk fiber was used to prepare a 10 cm × 10 cm silk nonwoven fabric. The carded fiber assembly was sprayed with distilled water for 10 min and pressed twice using a hot presser (HK 2008-1-5, Hankuk Industry Co., Gwangju, Republic of Korea) at 200 °C for 90 s to produce a silk nonwoven fabric [49]. Polyester nonwoven fabrics were placed on top and at the bottom of the silk nonwoven fabric during wetting and hot press treatments to prevent the silk nonwoven fabric from adhering to the hot presser plates. After hot-pressing, the silk nonwoven fabric was obtained by removing the polyester nonwoven fabrics. The code names of the silk nonwoven fabrics with varying sericin contents are listed in Table 1.

### 2.4. Measurement and Characterization

Photographic images of the silk nonwoven fabrics were obtained using a digital camera (iPhone 11 Pro, Apple Inc., Cupertino, CA, USA). The silk nonwoven fabrics were coated with Pt–Pd for morphological examination by field-emission SEM (FE-SEM, S-4800, Hitachi, Tokyo, Japan).

The yellowing index (YI) of the silk nonwoven fabrics was measured in CIE 1931 color space. CIE tristimulus (XYZ) values were based on the CIE standard illuminant C and the specular component excluded mode of the colorimeter (Konica Minolta, CM-700d Chroma Meter, Osaka, Japan). YI (ASTM method D1925) was calculated in accordance with Equation (3) [55].

(3) $$YI=\frac{\left(1.28X-1.06Z\right)}{Y}\times 100$$

To measure the porosity of silk nonwoven fabrics, they were immersed in a known volume of ethanol $V_{1}$ for 5 min. After the silk nonwoven fabrics were completely immersed and the ethanol permeated the nonwoven fabric sample, the total volume $V_{2}$ of the ethanol and silk nonwoven fabric was measured. The silk nonwoven fabric was removed from ethanol, and the residual ethanol volume ($V_{3}$) was measured. The porosity of the silk nonwoven fabric was obtained using Equation (4) [28,56]. This measurement was performed three times for each condition, and the average and standard deviation of the porosity of the silk nonwoven fabric was reported.

(4) $$\mathrm{Porosity}\;(\%)=\frac{V_{1}-V_{2}}{V_{2}-V_{3}}\times 100$$

The thickness of the silk filament was obtained from the SEM images using an image analysis program (DIMIS-PRO 2.0, Siwon Optical Technology, Anyang, Republic of Korea).

The molecular conformation and crystallinity of the silk nonwoven fabrics with varying sericin contents were determined using FTIR (Nicolet 380, Thermo Fisher Scientific, Waltham, MA, USA) using the ATR (Smart iTR ZnSe) method. The scan range, scan number, and resolution were 4000–650 cm^−1^, 32, and 8 cm^−1^, respectively.

The crystallinity index was calculated as the intensity ratio of the peaks occurring at 1620 and 1643 cm^−1^ in the FTIR spectrum, as represented by Equation (5) [57,58]. The FTIR measurements were performed seven times. The mean and the standard deviation of the crystallinity index were obtained from these seven FTIR measurements.

(5) $$\mathrm{Crystallinity}\;\mathrm{index}\;(\%)=\frac{A_{1620{cm}^{-1}}}{\;A_{1643{cm}^{-1}}+A_{1620{cm}^{-1}}}\times 100$$

where *A*_1620cm_^−1^ is the absorbance at 1620 cm^−1^ caused by the β-sheet crystallite (crystalline region), and *A*_1643cm_^−1^ is the absorbance at 1643 cm^−1^ attributed to random coil conformation (amorphous region).

The crystalline structures of the silk nonwoven fabrics were determined using a micro X-ray scattering system (D8 Discover, Bruker, Karlsruhe, Germany) using CuKα radiation. The irradiation conditions were 50 kV and 1000 μA, and the measurement time was 300 s.

The silk nonwoven fabrics were stored under standard conditions (20 °C and 65% relative humidity (RH)) for 24 h to determine their moisture regains, which were calculated using Equation (6) [58]. The dry weights of the silk samples were determined using a moisture-balance instrument (XM60, Precisa Gravimetrics, Dietikon, Switzerland).

(6) $$\mathrm{Moisture}\;\mathrm{regain}\;(\%)=\frac{Initial\;weight-Dry\;weight}{Dry\;weight}\times 100$$

DSC analysis was performed using a Thermal Analysis Instrument Q 10 (DS25, TA Instrument, New Castle, DE, USA) in the range of 60–270 °C at a scanning rate of 10 °C/min. The analysis was conducted under 50-mL/min nitrogen gas flow.

The mechanical properties of the silk nonwoven fabrics were evaluated using a universal testing machine (OTT-003, Oriental TM, Ansan, Republic of Korea). In the case of silk nonwoven fabric, the mechanical test was performed using a 20 kgf load cell. The extension speed and gauge length were 10 mm/min and 30 mm, respectively. The silk nonwoven fabric samples were cut into 50 mm × 10 mm pieces and preconditioned at 20 °C and 65% RH for more than 24 h. Seven nonwoven fabric samples were tested for each condition, and the average and standard deviation of the measurement results were obtained from the five results after the maximum and minimum values were excluded.

The effects of silk nonwoven fabrics on cytotoxicity were assessed using the CCK-8 assay. The silk nonwoven fabric was cut into 6 cm × 3 cm pieces. L929 mouse fibroblast cells (CCL-1) were provided by ATCC (USA) and grown in an RPMI1640 medium (Gibco) supplemented with 10% (*v*/*v*) fetal bovine serum and 1% (*v*/*v*) antibiotic–antimycotic solution. The L929 cells were incubated at 37 °C in a humidified 5% CO₂ atmosphere. When 80% confluence was observed, subculturing was conducted twice per week. In vitro cytotoxicity tests of the silk nonwoven fabric samples were conducted using an extraction method following ISO 10993-5. Before the extraction, each silk nonwoven fabric sample was sterilized with ethylene oxide gas. The extraction was performed by immersing the silk nonwoven fabric samples in the 6-mL RPMI1640 culture medium with gentle shaking at 37 °C for 24 h. Latex and HDPE, respectively, were used as the positive and negative controls. The cells were seeded into 96-well plates at a 1 × $10^{5}$ cells/mL ratio and incubated at 37 °C for 24 h in a 5% CO₂ atmosphere. The culture medium was then replaced with 100 µL/well of sample extracts. After 24 and 48 h of incubation, the extracts were discarded for the CCK assay, and CCK-8 solution was added to each well. After 1 h of incubation, the absorbance was measured at 450 nm. Subsequently, the cell viability of the silk nonwoven fabrics was obtained by Equation (7) [48,59].

(7) $$\mathrm{Cell}\;\mathrm{viability}\;(\%)=\frac{Absorbance\;of\;the\;test\;sample}{Absorbance\;of\;the\;control}\times 100$$

The cytotoxicity was also examined with a live/dead viability/cytotoxicity kit (L3224, Invitrogen, Waltham, MA, USA). The culture medium was replaced with 300 µL/well of the nonwoven fabric sample extracts. After 24 and 48 h of incubation, the extracts were discarded, and a staining solution was added to each well. After the incubation, the staining solution was removed, and the cells were observed by an inverted fluorescence microscope (IX83, Olympus, Tokyo, Japan).

## 3. Results and Discussion

### 3.1. Morphology of the Silk Nonwoven Fabric

Table 2 shows the external features of silk nonwoven fabrics after carding, wetting, and hot-pressing the silk fibers with varying sericin contents. Regardless of the sericin content, the preparation of silk nonwoven fabrics was possible. However, in the case of 0% sericin content (SNFS0), the structure was bulky, and silk fibers were loose. Since layers in this fabric were not bound tightly, they separated easily, even with a weak extensional force. As seen from Table 3, the cross-sectional structure of SNFS0 was also loose, whereas SNFS3.2 exhibited a thin and tight cross-sectional structure. This was due to the absence of a binder (sericin) in SNFS0. Moreover, the silk nonwoven fabrics were prepared by binding silk fibers by utilizing the binding character of sericin [27]. Accordingly, in the case of SNFS0, silk fibers could not be bound together because of the absence of a binder (i.e., sericin), resulting in a loose silk nonwoven fabric.

Unlike SNFS0, other silk nonwoven fabrics (SNFS3.2–SNFS24) exhibited tight and stable structures. Notably, SNFS3.2 exhibited a tightly structured fabric, indicating that 3.2% sericin content successfully bound silk fibers in fabricating silk nonwoven fabrics. This result is consistent with that of a previous report, where 2.6% sericin sufficed in fabricating silk/rayon nonwoven fabrics [48].

The morphologies of the silk nonwoven fabrics with varying sericin contents were observed, and the images are shown in Table 4. Unlike silk nonwoven fabrics prepared by reeling (winding) silk fibers in previous studies [27,47,48,49], the silk fibers in the fabrics prepared herein were arranged randomly. Considering that the silk fibers were arranged in a single direction using a hand carder, the silk fibers were expected to be arranged in a certain direction. Thus, the randomly arranged silk fibers in the fabrics in Table 4 were unexpected. This can be attributed to the original form of the silk samples. That is, silk fibers used herein were obtained from silkworm cocoons or degummed cocoons. Apparently, the isotropic arrangement of the silk fibers was maintained despite the carding process. Considering that silk nonwoven fabrics can be used in various applications, the fact that isotropic and anisotropic arranged silk nonwoven fabrics can be prepared by selecting a suitable preparation method (i.e., winding or carding) benefits the use of silk nonwoven fabrics in such applications.

The bulky structure of SNFS0 could be observed through scanning electron microscopy (SEM). Meanwhile, at sericin contents >3.2%, the morphological structure of the fabrics became compact. As the sericin content increased, the fabrics became more compact, and the silk fibers became tighter. This was due to the binding effect of sericin. That is, as the sericin content in the silk fibers increased, the fibers became swollen following wet treatment and deformed after hot-pressing, resulting in more compact and structured fabrics with smoother surfaces [27].

**Figure 2:**
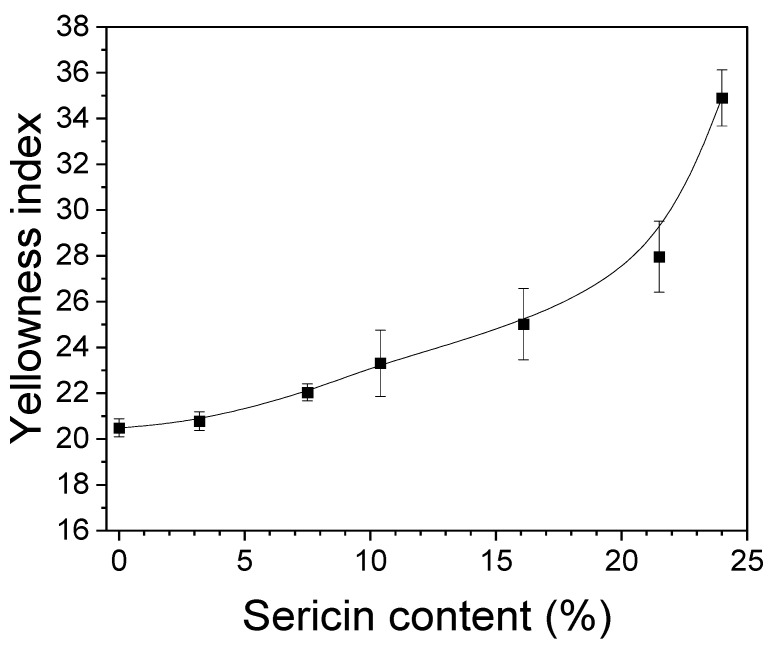
Effect of sericin content on the yellowness index of silk nonwoven fabrics (*n* = 3).

As seen from Table 2, the color of the silk nonwoven fabrics becomes yellowish with increasing sericin content. Hence, the yellowness index was measured to quantitatively examine the color change of the fabrics, and the results are shown in Figure 2. As the sericin content increased up to 21.5%, the yellowness index increased linearly, and it increased considerably to 24%. This confirmed the yellowing of the fabrics shown in Table 2 and indicated that sericin caused the yellowing of the silk nonwoven fabrics.

The yellowing was consistent with the results of previous reports [27,48,49,60]. For instance, Setoyama reported that the yellowing index of silk increases with increasing temperature because of the loss of hydroxyl amino acids due to the application of heat [61]. Particularly, silk sericin has higher amounts of hydroxyl amino acids, including serine (32%), aspartic acid (16.8%), and threonine (8%), as compared to silk fibroin [62,63]. This explains the increase in the yellowness index of fabrics with increasing sericin content in Figure 2.

Porous nonwoven fabrics have been widely used in cosmetics (e.g., mask packs) [15,57] and biomedical applications, including wound dressings [3,5] and membranes for guided regeneration [6,7,8,12] because they can hold water [30] and allow cell adhesion and proliferation [29,31,33] through their pores. Meanwhile, porosity determines the mechanical properties of porous materials [25,32]. Therefore, the porosity of silk nonwoven fabrics has been studied as an important structural factor [28,48,49].

**Figure 3:**
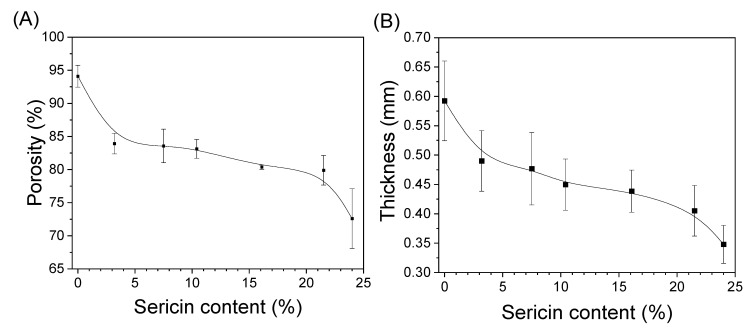
(**A**) Porosity (*n* = 3) and (**B**) thickness (*n* = 25) of silk nonwoven fabrics prepared with varying sericin contents.

Figure 3 shows the porosity and thickness of the silk nonwoven fabrics with varying sericin contents. The porosity of SNFS0 was 94.1%. However, porosity decreased with increasing sericin content; the porosity of SNFS24 was 72.7%. This result further confirms the increase in the compactness of the silk nonwoven fabrics with increasing sericin content, as shown in Table 2, Table 3 and Table 4. As shown in Figure 3B, the thickness of silk nonwoven fabrics showed a trend similar to that of the porosity of silk fabrics. This indicates that the decrease in porosity of silk fabrics is closely related to their thicknesses. The decreased thickness of the fabrics with increased sericin content was due to the increased binding effect of sericin. As discussed earlier, increased sericin content bound the fibroin fibers largely, which became more deformed, making the structure of the nonwoven fabrics denser. These results indicate that the porosity of silk nonwoven fabrics can be controlled by varying the sericin content.

### 3.2. Structural Characteristics of Silk Nonwoven Fabrics

**Figure 4:**
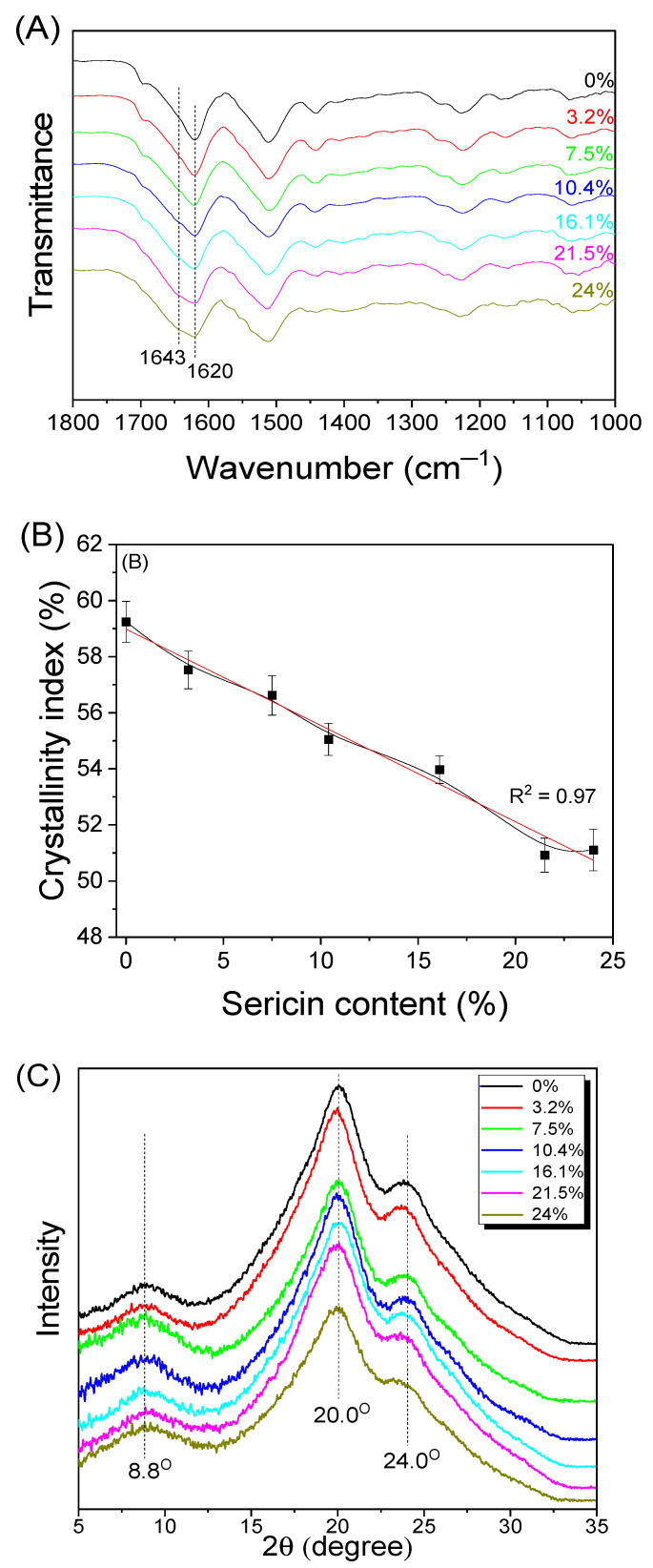
(**A**) Attenuated total reflectance–Fourier transform infrared spectra, (**B**) crystallinity indices, and (**C**) X-ray diffractograms of silk nonwoven fabrics with varying sericin contents.

Since the physical properties of silk are strongly affected by crystalline structures and molecular conformations [23,52,64], studies on these have been extensively conducted. Herein, Fourier transform infrared spectroscopy (FTIR) and X-ray diffraction (XRD) measurements were performed to examine the crystalline structures and molecular conformations of silk nonwoven fabrics; the results are shown in Figure 4.

SNFS0 showed a strong infrared (IR) absorption peak at 1620 cm^−1^ attributed to β-sheet crystallite [65,66]. As the sericin content increased, IR absorption at 1643 cm^−1^ attributed to random coil conformation increased constantly. Finally, SNFS24 showed a shoulder at 1643 cm^−1^. This indicated that the random coil conformation of silk fabrics increased with increasing sericin content. This was because the β-sheet crystallite of sericin was transferred to a random coil conformation when the sericin was exposed to the hot press at 200 °C [27,49]. The crystallinity index was calculated to quantitatively examine the molecular conformational change of the silk nonwoven fabrics depending on the sericin content, and the results are shown in Figure 4B. The crystallinity index of the fabrics exhibited a negative linear correlation with the sericin content ($R^{2}$ = 0.97), confirming the FTIR results in Figure 4A.

Figure 4C shows the results of XRD analysis on silk nonwoven fabrics. Regardless of the sericin content, all silk nonwoven fabrics showed XRD peaks at 8.8°, 20.0°, and 24.0° attributed to the β-sheet crystallite [48,67,68,69,70]. As the sericin content increased, the diffraction peak at 24.0° became less evident, indicating that the overall crystallinity of the silk nonwoven fabrics decreased. This result is consistent with that of the FTIR measurements.

**Figure 5:**
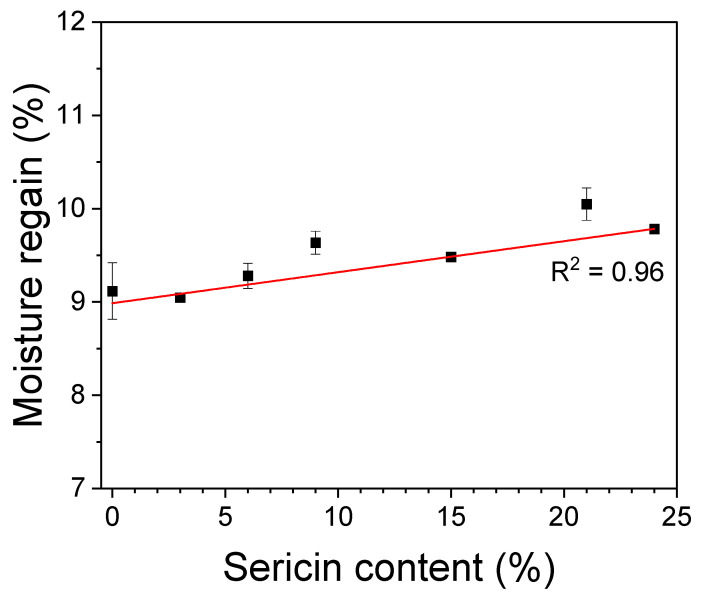
Moisture regains of silk nonwoven fabrics with varying sericin contents (*n* = 3).

The water absorption property of materials is important in cosmetic and biomedical applications. It prevents the drying of the wet ingredients of mask packs and helps in absorbing and holding the exudate in wound dressings, thereby healing wounds. Figure 5 shows the effect of sericin content on the moisture regain of silk nonwoven fabrics. The silk nonwoven fabrics exhibited moisture regain values ranging from 9–10%. As the sericin content increased, the moisture regain values of the fabrics increased slightly ($R^{2}$ = 0.96). The increase in moisture regain of the fabrics with increasing sericin content was attributed to the crystallinity and hydrophilicity of sericin. That is, a larger amorphous region (i.e., decreased crystallinity) facilitates higher water absorption and a correlation between these quantities was reported in previous studies [52,58]. Further, sericin is more hydrophilic than fibroin as it has higher contents of hydrophilic and polar amino acids [51]. Therefore, the silk nonwoven fabrics with increasing sericin contents naturally exhibit higher hydrophilicity.

**Figure 6:**
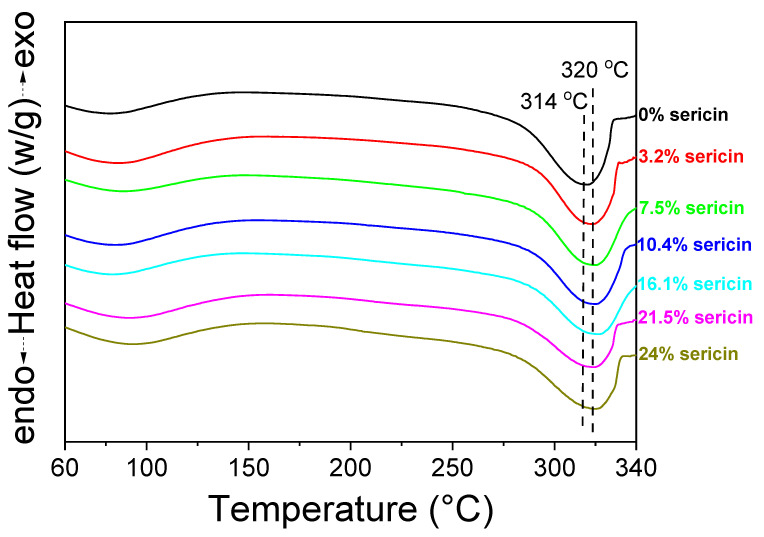
Differential scanning calorimetry thermograms of silk nonwoven fabrics with varying sericin contents.

The thermal behavior of the silk nonwoven fabrics with varying sericin contents was evaluated through differential scanning calorimetry (DSC), and the results are shown in Figure 6. SNFS0 showed an endothermic peak at ~314 °C attributed to the decomposition of fibroin [70,71]. However, in the cases with sericin contents of 3.2–24%, the endothermic peak was observed at 320 °C. Considering that the endothermic peak was due to the thermal decomposition of silk fibroin, the thermal decomposition temperature of the silk nonwoven fabrics did not change with the variation in the sericin content. SNFS0 had a lower decomposition temperature (i.e., 314 °C) than other silk fabrics (320 °C) due to the partial disruption of the crystalline region of fibroin during the degumming process. That is, when sericin was completely removed from silk by degumming, the molecular weight (MW) and crystallinity of fibroin were affected [46,72,73].

### 3.3. Mechanical Properties of Silk Nonwoven Fabrics

**Figure 7:**
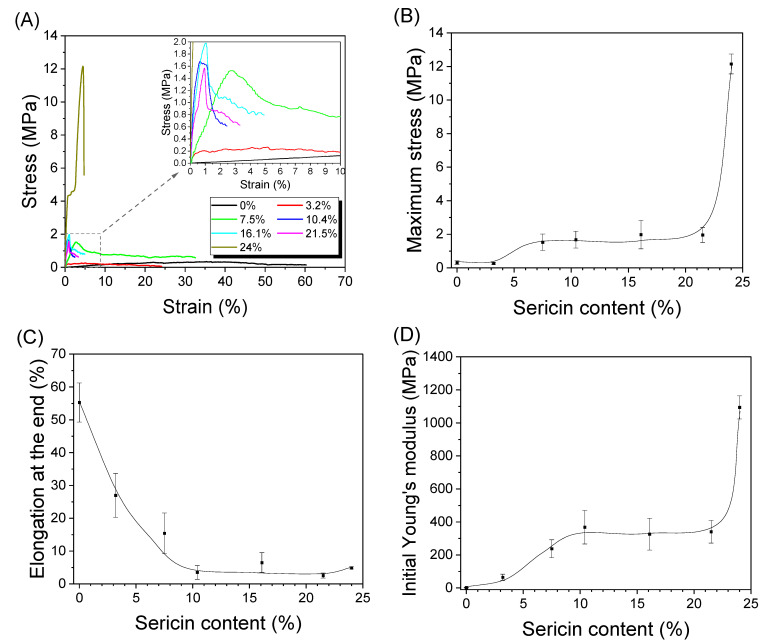
(**A**) Representative stress–strain curves, (**B**) maximum stress, (**C**) elongation at the end, and (**D**) initial Young’s modulus of silk nonwoven fabrics with varying sericin contents (*n* = 5).

Figure 7 shows the mechanical properties of the silk nonwoven fabrics with varying sericin contents. Without the addition of sericin, the silk nonwoven fabrics exhibited a maximum stress of 0.3 MPa and an elongation at the end of 55%. As the sericin content increased to 21.5%, the silk nonwoven fabrics exhibited a maximum stress of 2 MPa, and the elongation decreased to 2.5%. At a sericin content of 24%, the maximum stress increased to 12 MPa, but the elongation was unchanged. Overall, as the sericin content increased, the maximum stress increased, and the elongation at the end decreased. This trend was associated with the binding effect of sericin [27]. As the sericin content increased, the sericin in the silk fibers bound tightly with the neighboring silk fibers, leading to a stiffer nonwoven fabric. Further, the compact structure at higher sericin content (as shown in Table 4 and Figure 3) was responsible for the higher maximum stress of the fabrics. As discussed earlier, the surface crystallinity (FTIR-attenuated total reflection; ATR) and overall crystallinity (XRD) of the silk fabrics decreased with increasing sericin content. The increase in the maximum stress with increasing sericin content, despite the decrease in crystallinity, indicated that the binding effect of sericin dominated the effect of crystallinity in the fabrics.

The low maximum stress of the silk fabrics with 3.2% or less sericin content indicated that the binding force between the silk filaments was weak. Notably, the maximum stress (12 MPa) increased considerably at a sericin content of 24%. This may be due to the changes in compactness, porosity, and thickness, as shown in Table 4 and Figure 3.

### 3.4. Cell Viability of Silk Nonwoven Fabrics

**Figure 8:**
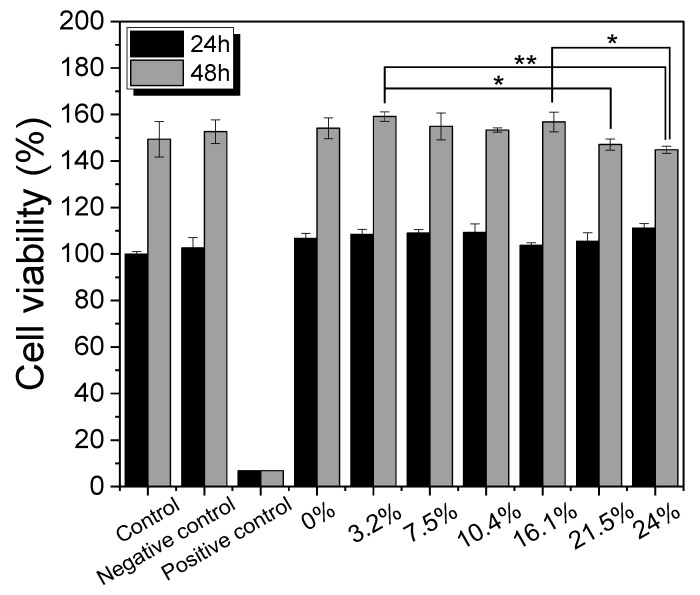
Cell viability of the silk nonwoven fabric with varying sericin contents (* *p* < 0.05, ** *p* < 0.01).

Since silk nonwoven fabrics can be used in biomedical and cosmetic applications, their cell viability was evaluated. The results are shown in Figure 8. In the case of 24 h incubation, regardless of the sericin content, all silk nonwoven fabrics exhibited similar or slightly higher cell viability than the control and negative control (i.e., >100%). That is, silk nonwoven fabric samples did not show significant differences among them. For reference, in the cell viability test, cell viability >80% was considered low cytotoxicity [74,75]. As the incubation duration increased to 48 h, the cell viability of all silk nonwoven fabrics increased up to >140%. Further, for an incubation time of 48 h, all silk nonwoven fabrics exhibited optimal cell viability comparable with that of the control and negative control, regardless of the sericin content. As for the effect of the sericin content, the silk fabric with sericin contents of 21.5% and 24% exhibited slightly lower cell viability than that of the other silk nonwoven fabrics. That is, the silk nonwoven fabric with 24% sericin content showed significant differences with 3.2% sericin content (** *p* < 0.01) and 16.1% sericin content (* *p* < 0.05) samples. In addition, the 21.5% sericin content sample showed a significant difference from the 3.2% sericin content sample (* *p* < 0.05). This slight decrease in cell viability with increasing sericin content was consistent with the results of a previous report [27].

The cell images of silk nonwoven fabrics with varying sericin contents shown in Table 5 further confirm the cell counting kit (CCK) test result shown in Figure 8. Regardless of the sericin content, all silk fabrics exhibited excellent cytocompatibility. The number of live cells of the silk fabrics at an incubation time of 24 h was comparable with that of the control and negative control and increased with increasing incubation time up to 48 h.

## 4. Conclusions

Herein, silk nonwoven fabrics were successfully prepared by carding silk fibers, and the effect of sericin content on the structural characteristics and properties of silk fabrics was investigated. As the sericin content increased, its binding effect increased, leading to more compact morphology of nonwoven fabrics with reduced porosity and thickness. The crystallinity and elongation of the fabrics decreased, and the moisture regain, and maximum stress increased with increasing sericin content. Regardless of the sericin content, all silk nonwoven fabrics exhibited excellent cytocompatibility. Further, the results indicated that the structure and properties of silk nonwoven fabrics could be manipulated by varying the sericin content, although the optimal cell viability of the fabrics was maintained. With advantages such as easy mass production and varied performances, new silk nonwoven fabrics prepared through carding are promising candidates for biomedical and cosmetic applications in the future.

## Figures and Tables

**Table 1:** Sample code and degumming conditions of silk nonwoven fabrics with varying sericin contents.

Sericin Content (%)	0%	3.2%	7.5%	10.4%	16.1%	21.5%	24%
Sample code of silk nonwoven fabric	SNFS0	SNFS3.2	SNFS7.5	SNFS10.4	SNFS16.1	SNFS21.5	SNFS24
Degumming ratio (%)	24.0%	21.5%	17.8%	15.2%	9.4%	3.2%	0%
Degumming method	Soap/ soda	HTHP	HTHP	HTHP	HTHP	HTHP	—
Degumming time	1 h	30 min	22 min	15 min	3 min	1 min	—

**Table 2:** Photographs of silk nonwoven fabrics with varying sericin contents. The white bar represents 1.0 cm length for reference.

SNFS0	SNFS3.2	SNFS7.5	SNFS10.4	SNFS16.1	SNFS21.5	SNFS24
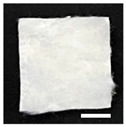	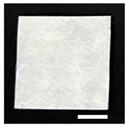	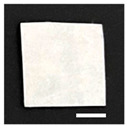	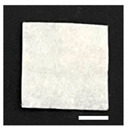	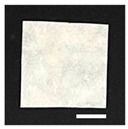	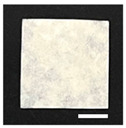	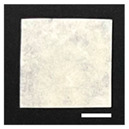

**Table 3:** Photographs of the cross-sections of silk nonwoven fabrics with 0% sericin content (SNFS0) and 3.2% sericin content (SNFS3.2). The white bar represents 1.0 cm length for reference.

SNFS0	SNFS3.2
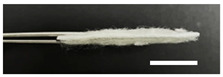	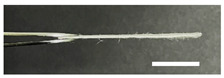

**Table 4:** Field-emission scanning electron microscopy images of silk nonwoven fabrics with varying sericin contents. The white bars represent 1.0 mm (low magnification) and 100 μm (high magnification) lengths for reference.

Magnification	Silk Nonwoven Fabric						
	SNFS0	SNFS3.2	SNFS7.5	SNFS10.4	SNFS16.1	SNFS21.5	SNFS24
**Low** **magnification**	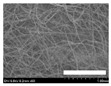	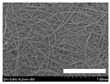	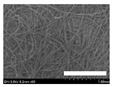	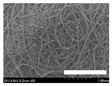	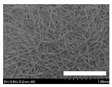	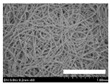	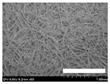
**High** **magnification**	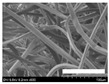	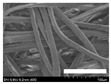	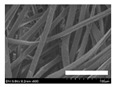	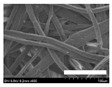	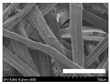	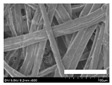	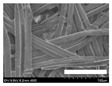

**Table 5:** Fluorescence images of the cell viability assay of the silk nonwoven fabric with varying sericin contents. The white bar represents 500 μm length for reference.

Incubation Time	Control	Negative Control	Positive Control	
24 h	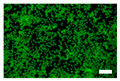	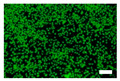	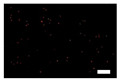	
48 h	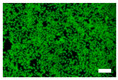	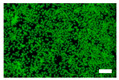	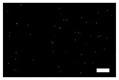	
**Incubation time**	**0% Sericin**	**3.2% Sericin**	**7.5% Sericin**	
24 h	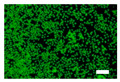	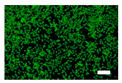	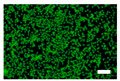	
48 h	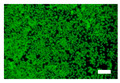	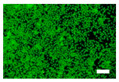	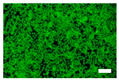	
**Incubation time**	**10.4% Sericin**	**16.1% Sericin**	**21.5% Sericin**	**24% Sericin**
24 h	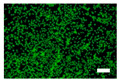	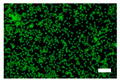	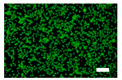	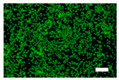
48 h	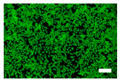	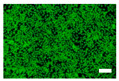	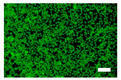	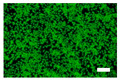

## Data Availability

The data presented in this study are available on request from the corresponding author.
